# Harnessing the Power of Data to Guide Local Action and End Tuberculosis

**DOI:** 10.1093/infdis/jix374

**Published:** 2017-11-06

**Authors:** Charalambos Sismanidis, Priya B Shete, Christian Lienhardt, Katherine Floyd, Mario Raviglione

**Affiliations:** 1 Global TB Programme, World Health Organization, Geneva, Switzerland

**Keywords:** Tuberculosis, surveillance, epidemiology, data use, programmatic action

The End TB Strategy approved by the World Health Assembly in May 2014 aims to end the global tuberculosis epidemic by 2035, with the ambitious targets of a 95% reduction of tuberculosis mortality, a 90% decline in tuberculosis incidence, and zero catastrophic costs for tuberculosis-affected households [1]. Despite recent advances in the diagnosis and treatment for tuberculosis, overall progress in reducing the incidence of tuberculosis and tuberculosis-related deaths, as well as the burden and costs associated with tuberculosis in the most affected communities, has been constrained by the inadequate implementation and scale-up of existing tools to detect, treat, and prevent tuberculosis [2]. A systematic review of World Health Organization–recommended interventions for tuberculosis prevention and care has demonstrated a lack of specific data on their effectiveness in real-world, programmatic settings [3]. This includes limited information on epidemiological impact and cost-effectiveness that are essential to inform decisions about scale up and the operationalization of evidence-based tuberculosis guidelines [3]. In 2016, more than 10.4 million new cases of tuberculosis were estimated to have occurred, with 6.1 million being reported to national surveillance systems, suggesting a failure in health systems to find, diagnose, and report approximately 4 million tuberculosis cases [2]. Country-level analyses of available epidemiological, health systems, and health-seeking behavior data can provide useful insights into where exactly these losses are observed along the patient pathway of tuberculosis care [4]. This approach allows for translating existing national- and subnational-level data into programmatic gaps that promote more favorable outcomes relevant to the care of tuberculosis patients, such as early case detection, reduced loss to follow-up of case patients before they start treatment, improved treatment outcomes, and decreased levels of mortality.

Articles published in this supplement illustrate how the analysis of patient pathways to tuberculosis care can identify the programmatic gaps in the implementation of key interventions for tuberculosis detection, care, and prevention. The importance of community referral networks for more effective detection of drug-sensitive tuberculosis in Ethiopia and the Philippines is highlighted [5, 6]. The role of bacteriological confirmation and notification of cases in Pakistan and Indonesia, where initial care-seeking behavior brings most individuals to the private sector, is identified as a key area to address [7, 8]. These analyses and their results highlight the need to integrate patient-centred approaches with the interpretation, monitoring, and evaluation of tuberculosis surveillance and, where necessary, survey data in national tuberculosis policy decision making. To reach the ambitious targets of the End TB Strategy, strengthened surveillance systems, patient-centred analyses, and intensified implementation research are essential to operationalize and optimize existing and novel interventions for tuberculosis prevention and high-quality care that address programmatic gaps and barriers faced by patients.

Improving the patient pathway of tuberculosis care requires a multifaceted approach that starts with the gap identification and description. This can be achieved through the analysis and use of available quality surveillance and survey data, both at national and subnational levels, and the optimal implementation of a package of complementary tools to synthesize and interpret these data for use in informing programmatic local action. This package of tools includes, but is not limited to, tuberculosis program reviews, tuberculosis epidemiological and impact reviews, diagnostic and patient pathway to tuberculosis care analyses, and mathematical modeling projections for the allocative efficiency of interventions and the expected impact they can have on the tuberculosis burden. Findings from this package of tools need to be translated into solutions for addressing barriers to reaching care, explain why these gaps exist, and demonstrate how to close them such as through implementation research, for example. By providing a framework (Figure 1) to national stakeholders that starts from the identification and description of gaps and then translates gaps into activities and targeted interventions, we can improve program performance and healthcare delivery by restructuring health systems and overcoming barriers to care in a context-specific and evidence-based manner.

**Figure 1.**
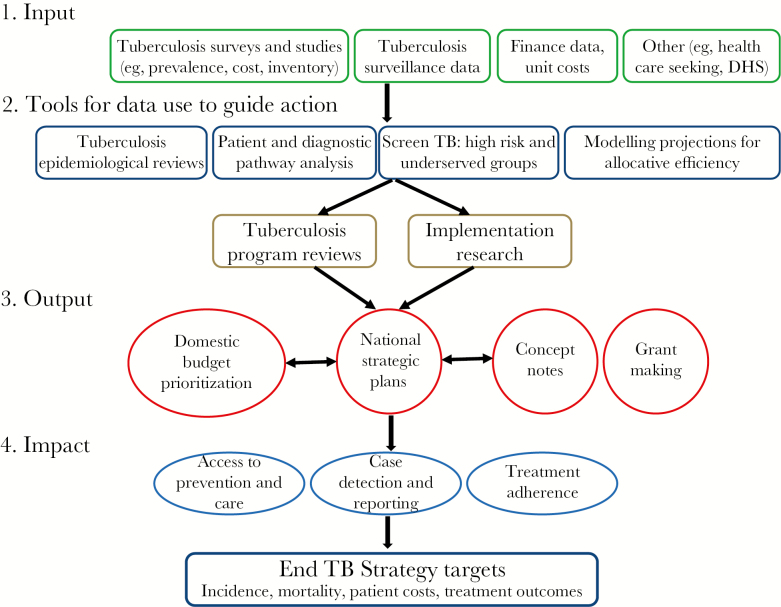
Framework for use of data and implementation of tools to identify and address gaps in tuberculosis prevention and care and contribute toward reaching the targets of the End TB Strategy.

Tuberculosis epidemiological reviews offer a systematic description and assessment of the surveillance systems in place to monitor tuberculosis cases and deaths, including the compilation of national and subnational tuberculosis surveillance data to study and interpret time trends, as well as collate data on determinants of tuberculosis [2]. These data often lead to the identification of corrective actions required to strengthen surveillance systems and improve the direct measurement of the tuberculosis burden. Patient and diagnostic pathway analyses identify and describe the gaps and barriers all along the cascade of care, mapping epidemiological data to the patient experience [9]. The Screen TB tool offers insights into target prioritization and strategy selection for tuberculosis screening among groups that are either at high risk of developing tuberculosis disease and/or are underserved and vulnerable [10]. Finally, mathematical modeling projections allow an investigation into the impact that available interventions could have on the tuberculosis burden [11].

The current global status of available key surveillance and survey tuberculosis data is shown in Figure 2. The best approach to estimating tuberculosis incidence, a key indicator of the End TB Strategy, is through robust routine surveillance of tuberculosis case notifications. In settings where universal health coverage is available and efforts are made to understand and quantify the gaps of the surveillance system, notifications are considered a proxy for incidence (Figure 2A) [2]. The major reasons why cases are missing from routine surveillance of notification data include laboratory errors, lack of notification of cases by public and private providers, failure of people accessing health services with suggestive symptoms to be identified as potential tuberculosis cases, and lack of access to health services [2]. Tuberculosis mortality, another key indicator of the End TB Strategy, is best captured from vital registration systems with high coverage and accurate recording of cause of death according to the latest revision of the International Classification of Diseases (Figure 2B) [2]. In addition to routine surveillance, special studies and surveys carried out on a post hoc basis also provide empirical measurements of the tuberculosis burden. A true revolution in the availability of robust data on tuberculosis burden has been observed since 2009 in the form of national tuberculosis prevalence surveys among the general population, with 23 countries implementing a survey (Figure 2C) [2]. Meanwhile, the Global Project on Anti-TB Drug Resistance Surveillance has been systematically collecting and analyzing data on drug resistance worldwide since the mid-1990s (Figure 2D) [2]. More recently, new types of tuberculosis studies are also being implemented and are providing important data: tuberculosis inventory studies that measure tuberculosis underreporting, and cost surveys that measure the percentage of tuberculosis cases and their households that face catastrophic costs as a result of tuberculosis disease [2].

**Figure 2.**
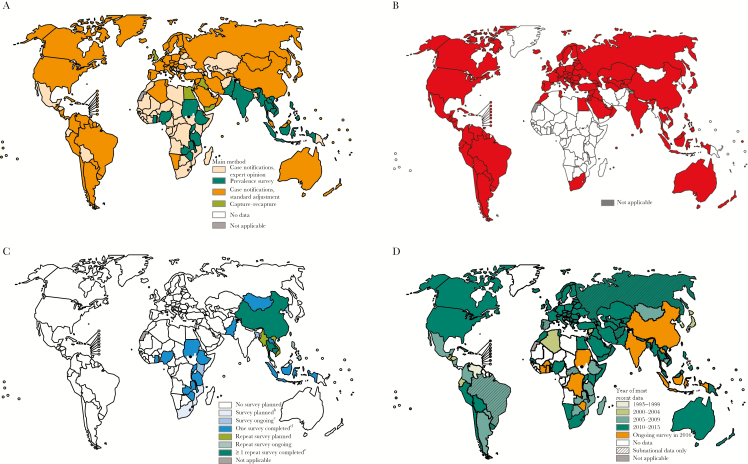
Current methods to monitor levels of, and trends in, tuberculosis incidence (*A*), tuberculosis mortality in HIV-negative people estimated using measurements from vital registration systems and/or a mortality survey (*B*), status of empirical measurements of tuberculosis burden with national prevalence surveys (*C*), and surveillance of antituberculosis drug resistance (*D*).

As described in the articles in this supplement, leveraging these rich and varied data sources and synthesizing them into a patient-centred analysis of health system performance can clearly identify and describe specific programmatic gaps in tuberculosis care at both national and subnational levels. What next then? The usefulness of this gap analysis is only as strong as its ability to direct us to solutions that ultimately improve tuberculosis patient outcomes. Implementation research (IR) can provide a necessary, systematic approach to explaining why these gaps exist, addressing patient and provider barriers to care, and using systematic frameworks to design evidence-based approaches on how to overcome programmatic obstacles.

Implementation research is designed to find ways to close evidence-practice gaps—gaps between what is recommended and what actually occurs in routine tuberculosis practice. By understanding the difference between current practice in tuberculosis care and the ideal in terms of underlying behavioral and structural contexts, and the outcome gap, the impact on tuberculosis and overall health outcomes as a result of implementation of evidence-based care, IR seeks to identify and make concrete targets for improved implementation strategies. An approach rooted in IR presents a step-wise model for programs and other key stakeholders in planning, designing, and evaluating intervention deployment [12]. In the past, the majority of evidence-based healthcare interventions, including tuberculosis care, have been adopted in an ad hoc manner, with little basis and evidence on how to optimally scale up. Implementation research counters this tendency by providing a framework for engaging stakeholders in planning, considering both structural and strategic contexts that could affect program implementation, conducting formative research necessary to identify the behavior change required to improve implementation, designing interventions that target those behaviors, and setting up robust monitoring and evaluation systems that provide evidence of both implementation strategy uptake and intervention effect [13]. The result is that programs and stakeholders have a clearer sense of what worked, what did not work, and why.

The benefits of combining available gap identification tools with IR are potentially game changing for tuberculosis control. Findings from the optimal implementation of the available package of tools allow national tuberculosis programs to develop national and subnational strategic plans (NSPs) for tuberculosis that take into account the context-specific epidemiology, health system, and tuberculosis determinants. These NSPs can then be leveraged to attract support from both domestic and international funding sources, either as part of national research and program funds or through proposals to funding institutions. A recent study indicates that country programs are hesitant to request such funds, even when the opportunity exists [14]. This is likely caused by a lack of a systematic approach to synthesizing context-specific data into meaningful action with patient and public health impact. By using this package of tools with IR, one can provide a framework to programs and stakeholders that identifies gaps, translates gaps into research questions, provides a framework to design pilot projects to answer these questions, and evaluates the impact of an implementation strategy to obtain meaningful context-specific data. This approach is expected to provide the basis for rapid and appropriate scale-up, implementation, and dissemination of new tools and interventions, which are critical for achieving short- (2020) and medium-term (2025) End TB Strategy targets, better tuberculosis care delivery, less suffering, and reduced transmission.

The tuberculosis world is currently witnessing a real revolution in the availability of surveillance and survey data at both national and subnational levels and a package of complementary tools that can harness the power of those data and translate them into policy, planning, advocacy, and programmatic action. Optimal implementation of these tools provides a real opportunity for progress toward reaching the ambitious targets of the End TB Strategy, one we cannot afford to miss.

## Notes

***Supplement sponsorship.*** This work is part of a supplement sponsored by the Bill & Melinda Gates Foundation.

***Potential conflicts of interest.*** All authors: No reported conflicts of interest. All authors have submitted the ICMJE Form for Disclosure of Potential Conflicts of Interest. Conflicts that the editors consider relevant to the content of the manuscript have been disclosed.
